# Crowdsourcing to Reduce Stigma Around HIV testing Among Adolescents and Young Adults in Kazakhstan

**DOI:** 10.1093/eurpub/ckac131.343

**Published:** 2022-10-25

**Authors:** G Mergenova, D Gryazev, A Kuskulov, Y Sun, S Landers, A Tolegenova, V Gulyayev, P Gulyayev, A Davis, JasSpark Study Research Group

**Affiliations:** 1 Global Health Research Center of Central Asia, Almaty, Kazakhstan; 2 School of Social Work, Columbia University, New York, USA; 3 Epidemiology, Asfendiyarov Kazakh National Medical University, Almaty, Kazakhstan

## Abstract

**Background:**

New cases of HIV infection are increasing among adolescents and young adults (AYA) in Kazakhstan, and now account for over 23% of all HIV infections. Testing is key to reducing new infections, yet HIV-related stigma is a major barrier. Crowdsourcing contests could engage AYA in the development of interventions and increase the likelihood that an effective approach will be developed to reduce stigma and increase HIV testing among Kazakhstan AYA.

**Objective:**

To determine the response to a crowdsourcing approach to collect media content for the development of an intervention focused on HIV testing stigma reduction among AYA in Kazakhstan.

**Methods:**

To organize the crowdsourcing contest we organized a community steering committee; developed platforms to solicit crowd input; engaged AYA to contribute ideas through social media and in-person events. We present data on social media use and contributions.

**Results:**

The contest website was visited by 2,893 people, and 76 AYA submitted 91 works for the contest from all over Kazakhstan. Most AYA (60%) visited the website of the contest via social media. Types of submitted content for the contest included video: 31 (34.0%), pictures: 29 (31.8%), text: 24 (26.3%), and other types of content including a chatbot, online game, and website: 7 (7.6%). Fifty (65.7%) participants were between 13-19 years, and 26 (34.2%) were between 20-29 years. Thirteen (17.1%) submissions were in Kazakh, and the rest in Russian 63 (82,9%). A number of submissions required further guidance due to a lack of understanding about stigma, accurate facts, and copyright issues.

**Conclusions:**

AYA engaged in a crowdsourcing contest and used multiple modalities/types of content, from younger and older AYA, in both Kazakh and Russian, and representation from most regions of the country. Providing clearer guidance about facts and stigma may be helpful prior to submission.

**Key messages:**

• Crowdsourcing approach has a potential to engage adolescent and young adult into development of various types of media content focused on public health issues, such as HIV testing stigma reduction.

• Adolescents and young adults may need additional information and guidance regarding complex concept of stigma around HIV testing.

